# Evaluation of pharmacist-led telemedicine medication management for hypertension established patients during COVID-19 pandemic: A pilot study

**DOI:** 10.3389/fpubh.2022.1091484

**Published:** 2022-12-16

**Authors:** Xiaoye Li, Jialu Hu, Yao Yao, Chengchun Zuo, Zi Wang, Xiaoyu Li, Qianzhou Lv

**Affiliations:** ^1^Department of Pharmacy, Zhongshan Hospital, Fudan University, Shanghai, China; ^2^Department of Cardiology, Zhongshan Hospital, Fudan University, Shanghai, China

**Keywords:** telemedicine medication management, usual care, hypertension management, COVID-19 pandemic, blood pressure

## Abstract

**Aim:**

To evaluate the impact of a telemedicine medication management service in patients with hypertension.

**Methods:**

Participants were allocated to either a telemedicine service (*N* = 173) or usual care (UC) (*N* = 179). The primary outcome was blood pressure (BP) reduction from baseline to the 6-month follow-up visit, the proportion of the target BP achievement, overall adherence to prescribed medication as well as a composite of non-fatal stroke, non-fatal myocardial infarction and cardiovascular death.

**Results:**

At 6 months, BP was controlled in 89.6% (*n* = 155) of intervention patients and 78.8% (*n* = 141) of UC patients (OR = 1.14, 95% CI = 1.04–1.25, *P* = 0.006), giving a mean difference of −6.0 (−13.0 to −2.5 mmHg) and −2.0 mmHg (−4.0 to −0.1 mmHg) in SBP and DBP, respectively. 17.9% (*n* = 31) of the patients in the intervention group were non-adherent with medications, compared with 29.1% (*n* = 52) in the UC group (*P* = 0.014). The composite clinical endpoints were reached by 2.9% in the intervention group and 4.5% in the control group with no significant differences (OR = 1.566, 95% CI = 0.528–4.646).

**Conclusion:**

Telemedicine medication management for hypertension management had led to better BP control and medication adherence improvement than UC during COVID-19 epidemic, resulting in a reduction of overall adverse cardiovascular events occurrence.

## 1. Introduction

The COVID-19 pandemic has dramatically affected and overloaded healthcare systems across the globe and strained healthcare resources on many levels (1, 2). Services like medication counseling and care proved to be challenging for overloaded medical practitioners to provide (3). According to the 2018 Report on Cardiovascular Diseases in China, hypertension was an important public disease burden in China with 245 million patients (prevalence 23.2%) (4). Notwithstanding, the disease control was still suboptimal, well contributing to high risk of adverse cardiovascular outcomes, which requires long-term treatment with anti-hypertensives to control blood pressure (BP) (5). Appearing in person in clinic for routine hypertension monitoring and follow-up often exposed elderly and vulnerable patients to infectious risk. Telemedicine offers the prospect of remote management of BP for vulnerable individuals while avoiding the risks of in-person care in a pandemic (6, 7).

It was challenging to provide routine medication management for patients with hypertension and other chronic cardiovascular diseases during the pandemic (8, 9). To reduce the risk of infectious exposures, non-contact treatment models have been advocated in some settings, including China. The pharmaceutical department of the Zhongshan Hospital has built an efficient telemedicine pharmaceutical service model which leverages remote communication methods such as WeChat or telephone, to improve the service quality, reduce the risk of infection, and ensure the safety of patients (10). Telemedicine, by limiting person-to-person contact, might reduce the possibility of viral transmission, and offer the possibility of more timely care for chronic diseases. By leveraging pharmacist input, the telemedicine approach enables rapid remote review and evaluation of medication lists, indications, dosing particulars, storage methods, precautions and drug interactions.

Patients with adequate clear medication information are better equipped to make informed choices about managing their anti-hypertensives, and potentially might have more incentive and support to adhere to their prescribed medications. Therefore, this study aimed to evaluate the pharmaceutical telemedicine care service in patients with existing hypertension, as compared to usual care (UC).

## 2. Methods

### 2.1. Study design and patient information

This prospective single-site cohort study was designed to compare a telemedicine intervention to usual care for people with hypertension seeking care at the Zhogshan Hospital (Shanghai, China) between January 2021 and June 2021. In our study, hypertension was defined as an average office systolic BP of >140 mmHg or an average office diastolic BP of >90 mmHg or self-reported use of antihypertensive medication in the past 2 weeks according to the American Hypertension Management Guidelines (11). Patients with uncontrol hypertension were finally enrolled. Integrated care, such as health screenings, providing patient education and modifying medication regimens under collaborative practice agreements, was available for all patients who were diagnosed as hypertension including antihypertensive therapy and inconsistent approaches to cardiovascular risk reduction. All drugs were prescribed as single doses by pharmacists under the doctor's supervision. For follow-ups, BP was measured during outpatient clinic visits by specialized cardiologists or other certified specialists.

All patients involved met the following eligibility criteria: (1) age ≥ 18 years; (2) high risk for cardiovascular diseases in terms of diabetes, dyslipidemia, smoking, poor diet, and obesity; and (3) primary hypertension prescribed with antihypertensive drugs/daily. The main exclusion criteria included the following: (1) secondary hypertension; (2) severe renal dysfunction (estimated glomerular filtration rate [eGFR] <30 ml/min^−1^·1.73 m^2^); and (3) unable to communicate *via* WeChat or phone. This study was approved by the Ethics Committee of Zhongshan Hospital (Approval Number: B2021-021R). Written informed consent was signed by all participants before the commencement of the clinical studies.

### 2.2. Study procedure

#### 2.2.1. Procedure

The eligible participants were identified from clinical codes recorded in the electronic medical record system and invited to learn about this clinical study. Written informed consent for participation was obtained before the participants were determined to be eligible and we collected their baseline information *via* electronic health records. The BP was measured by standard mercury sphygmomanometers in a sitting position. At least two BP measurements should be taken in the sitting position, spaced 1–2 min apart and the average value was used for diagnosis (12). Consecutive participants were allocated to receive either the pharmacist-led telemedicine care and follow-up service or UC according to patients' demand and willingness.

The medication decision on patients' drugs was performed by clinicians' discretion throughout the whole study procedure. The participating pharmacists reviewed the medication for BP control and perform an individualized medication titration plan for the enrolled participants after allocation. Three and six months after allocation, scheduled follow-up appointments were performed to measure and record BP for attended participants for both groups.

#### 2.2.2. Pharmacist-led telemedicine pharmaceutical intervention

Continuous participants were assigned to telemedicine pharmaceutical intervention or UC group according to patients' demand and willingness. In the intervention group, there were five cardiovascular pharmacists in total who provided the interventions, mainly including the administration time and dosage of antihypertension drugs, the management of adverse reactions, the drug interactions and BP monitoring, which as indicated in Supplementary Figure 1. The antihypertension medication guidance was listed in Supplementary Table 1. Firstly, medication review was established to collect participants' demographic and clinical factors, and their understanding of current medication status. Then, intervention was conducted based on medication guidance including educational materials and individualized pharmacotherapy.

The participants were well trained in: (i) reviewing how to use a mercury sphygmomanometer to measure their BP at home; (ii) visiting the Web-based dashboard through smartphone or WeChat, entering the personalized goals, and getting to know how to enter and view their data; and (iii) developing a personalized BP management plan (e.g., frequency of contacts for check-ins, goal-setting, and data upload) informed by the baseline home BP measurements. Then they were asked to: (i) measure their BP at home use a mercury sphygmomanometer, while sitting, after a rest period of at least 10 min; (ii) send their BP data report to the researcher through smartphone or WeChat; and (iii) set BP targets weekly. 2 The role of the pharmacist in this study was to make medical intervention or lifestyle recommendations according to the BP data and description retrieving. Participants could withdraw from the study at any time.

#### 2.2.3. Pharmacist-led usual care

Participants who were allocated to UC group were not provided with an online drug counseling platform, but obtained online access for web information on hypertension control including classification and causes of hypertension, guidance for the hypertension management in terms of lifestyle improvement and medication adjustments. The participants received routine services by referring to an outpatient clinic for hypertension care which was typically composed of BP measurements for titrate drugs and antihypertensive adjustments to maintain target BP.

### 2.3. Data collection and follow-ups

The demographic information and baseline assessments including age, sex, body mass index (BMI), complications, and initial BP were obtained through electronic medical recorder (EMR). Laboratory measurements including serum sodium, potassium, creatinine, estimated glomerular filtration rate, and concomitant medication record were collected from EMR.

For each eligible participants, follow-up controls were scheduled every 3 months, up to at least 6 month. BP measurements were performed at the screening visit. Physicians provided medication related interventions to make every reasonable effort to control BP in accordance with international and local hypertension management guidelines. The medication related interventions were classified into 8 categories by pharmacist in this study, including stopped therapy, side effect, adherence improvements, costs, drug-drug interaction, liver function, renal function and inappropriate doses. Other than this, duration for the first outpatient revisit and the time to first or recurrent cardio-cerebral vascular events were recorded throughout follow-ups.

### 2.4. Adherence to antihypertensive therapy

Adherence to antihypertensive therapy was estimated by the self-report method at the point of 6-months follow-up *via* telephone interview or clinic visit (13, 14). Specifically, medication adherence was measured by proportion of days covered (PDC), defined as the number of days patients taking prescriptions divided by the interval of observation period. PDC above 80% was considered as full or high adherence (15). Conversely, the patient was considered to be non-adherent when he reported omitting dose of the medication, or making errors in dosage or frequency, or if he interrupted treatment. Reasons for medication non-adherence were recorded.

### 2.5. Clinical outcomes

The primary outcome was systolic and diastolic BP and heart rate reduction from baseline to the 6-month follow-up visit, the proportion of the target BP achievement, overall adherence to prescribed medication as well as a composite of non-fatal stroke, non-fatal myocardial infarction and cardiovascular death.

The secondary outcomes were adherence and persistence to antihypertensive agents, antiplatelet agents, lipid-lowering agents, proton pump inhibitor (PPI) and antiarrythmic drugs and cumulative incidence of any cardio-cerebral vascular event.

### 2.6. Sample size consideration

A sample size of 140 consecutive participants per group were required to have 90% power according to previously reported literature (16). The level of the statistical significance test (Class I error rate α) is 0.05 (Using two-sided inspection), the statistical effect is 90% (Class II error rate β = 0.1), and the sample size is estimated using PASS 11 statistics software. To accommodate an anticipated dropout rate of 10%, 308 participants were enrolled to achieve 280 evaluable participants.

### 2.7. Statistical analysis

This clinical study is a single-center and prospective cohort study, whose primary endpoint was to detect a difference of systolic and diastolic BP between the telemedicine pharmaceutical intervention and usual care group. The baseline characteristics were compared between intervention and usual groups by *t*-tests and χ^2^ tests/Fisher's precision probability test where appropriate. The comparison including the changes of SBP/DBP/heart rate (presented as means ± standard deviations) to baseline were analyzed by *t*-tests; the proportion of the target BP achievement, adherence with anti-hypertensive medications and medication related interventions were expressed with frequencies or percentages n (%) and compared with χ^2^/Fisher's precision probability test. Kaplan–Meier curves were constructed to compare the duration for the first outpatient revisit and the time to first or recurrent cardio-cerebral vascular events.

A two-sided *P*-value was used to determine significance (threshold, *P* < 0.05). Statistical analysis was performed using SPSS (IBM SPSS Statistics 22.0) and Prism 5 (GrandPad Software). A *P*-value of 0.05 was considered to be the threshold for statistical significance.

## 3. Results

### 3.1. Baseline characteristics of study population

**Figure 1:**
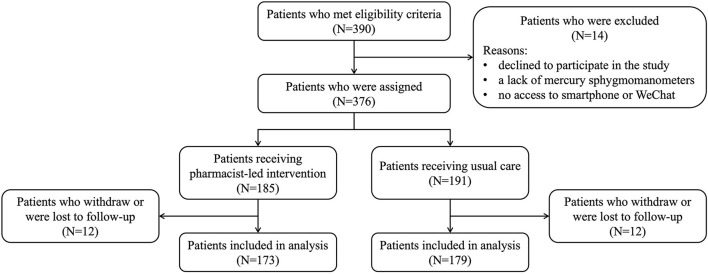
The flowchart of study screen and selection.

After screening for eligibility, a total of 390 patients consented to participate in this study. The whole recruitment progress of the study and the exclusive reasons were presented in Figure 1. 14 patients declined to take part in this study and gave their reasons for the lack of mercury sphygmomanometers and no access to smartphone or WeChat. The remaining 376 (96.4%) participants were divided into intervention (*N* = 185) and usual care group (*N* = 191). During the whole recruitment period, 12 in the intervention and 12 in the usual care group were subsequently withdrawn due to the withdraw and follow-up failure of the study. The adherence rate to intervention and usual care was 93.5 and 93.7% throughout whole study.

There were 173 subjects in the intervention group and 179 subjects in the UC group at the end of the follow-up periods. No notable differences between the group were found regarding baseline demographics, clinical characteristics and laboratory indicators of the population (Table 1). Most participants were male (62.2%) with a mean age of 68.10 ± 8.82 years. The mean baseline clinical BP for intervention group and usual care group were 151.47/88.45 and 150.77/88.83 mmHg respectively.

**Table 1:** Baseline demographic and clinical factors between intervention and UC groups.

	**Intervention (*N* = 173)**	**UC (*N* = 179)**	***P*-value**
**Baseline characteristics**			
Gender, male; *n* (%)	102 (59.0%)	117 (65.4%)	0.215
Age, years; mean (SD)	68.28 ± 8.50	67.93 ± 9.15	0.715
BMI, kg/m^2^; mean (SD)	24.77 ± 3.21	24.25 ± 3.15	0.124
Smoking (%)	26 (15.0%)	29 (16.2%)	0.762
Alcohol (%)	17 (9.8%)	19 (10.6%)	0.807
SBP, mmHg; mean (SD)	151.47 ± 7.27	150.77 ± 8.16	0.394
DBP, mmHg; mean (SD)	88.45 ± 3.60	88.83 ± 4.36	0.365
Heart rate; mean (SD)	76.40 ± 8.96	76.51 ± 10.36	0.917
**Comorbidities**			
Hyperlipidemia (%)	60 (34.7%)	52 (29.1%)	0.257
Diabetes (%)	56 (32.4%)	48 (26.8%)	0.254
Atrial fibrillation (%)	7 (4.0%)	8 (4.5%)	0.844
CHD, (%)	16 (9.2%)	17 (9.5%)	0.936
Liver dysfunction, (%)	15 (8.7%)	15 (8.4%)	0.922
Heart failure, (%)	37 (21.4%)	36 (20.1%)	0.768
stroke/TIA, (%)	30 (17.3%)	37 (20.7%)	0.426
PAD, (%)	6 (3.5%)	6 (3.4%)	0.952
**Laboratory tests**			
ALT, IU/L; median [IQR]	20.00 [15.00–31.00]	24.00 [17.00–30.00]	0.603
Hb, g/L; mean (SD)	136.08 ± 16.56	134.99 ± 19.03	0.558
PLT, *10^9^/L; mean (SD)	191.34 ± 55.45	185.85 ± 55.76	0.355
eGFR, mL/min/1.73 m^2^; median [IQR]	77.00 [68.00–86.00]	76.00 [67.00–83.00]	0.830
LDL-C, mmol/L; mean (SD)	2.20 ± 0.83	2.30 ± 0.88	0.269
HbA1c; mean (SD)	6.10 ± 0.84	5.99 ± 0.98	0.245
APTT, s; mean (SD)	31.38 ± 6.10	30.82 ± 5.31	0.353
PT, s; mean (SD)	12.86 ± 2.36	12.67 ± 2.58	0.460
TT, s; median [IQR]	18.00 [17.25–18.95]	17.60 [17.00–18.50]	0.925
FIB, mg/dL; median [IQR]	264.00 [228.25–310.25]	275.00 [225.50–319.75]	0.941
D-D, mg/L; median [IQR]	0.19 [0.06–0.44]	0.23 [0.19–0.31]	0.318
INR; median [IQR]	0.98 [0.93–1.10]	0.99 [0.96–1.06]	0.260
CK-MB, U/L; median [IQR]	13.44 ± 4.30	13.35 ± 4.68	0.855
NT-proBNP, pg/ml; median [IQR]	372.30 [178.45–1000.60]	337.70 [101.40–885.90]	0.732
>**Concomitant medication**			
CCB, (%)	92 (53.2%)	91 (50.8%)	0.660
ACEI, (%)	67 (38.7%)	68 (38.0%)	0.887
ARB, (%)	47 (27.2%)	48 (26.8%)	0.941
β-receptor antagonists, (%)	64 (37.0%)	71 (39.7%)	0.606
Diuretics, (%)	36 (20.8%)	28 (15.6%)	0.209
Oral antiplatelet, (%)	54 (31.2%)	50 (27.9%)	0.500
Lipid-lowering agent, (%)	93 (53.8%)	94 (52.5%)	0.815
Anti-arrythmic agent, (%)	15 (8.7%)	14 (7.8%)	0.772
PPI, (%)	18 (10.4%)	18 (10.1%)	0.914

Quantitative variables are shown as mean and standard deviation (SD). BMI, body mass index; SBP, systolic blood pressure; DBP, diastolic blood pressure; CHD, coronary heart disease; PAD, peripheral artery disease; ALT, alanine aminotransferase; Hb, hemoglobin; PLT, platelet count; eGFR, estimated glomerular filtration rate, calculated with the CKD-EPI equation; LDL-C, low-density lipoprotein cholesterol; HbA1c, glycosylated hemoglobin type A1c; APTT, activated partial thromboplastin time; PT, prothrombin time; TT, thrombin time; FIB, fibrinogen; D–D, d–dimer; INR, international normalized ratio; CK-MB, creatine kinase-MB; NT-proBNP, N-terminal pro-B-type natriuretic peptide; CCB, calcium channel blocker; ACEI, angiotension converting enzyme inhibitors; ARB, angiotensin receptor blocker; PPI, proton pump inhibitors.

### 3.2. Effect of intervention (compared with UC) on BP and HR

**Figure 2:**
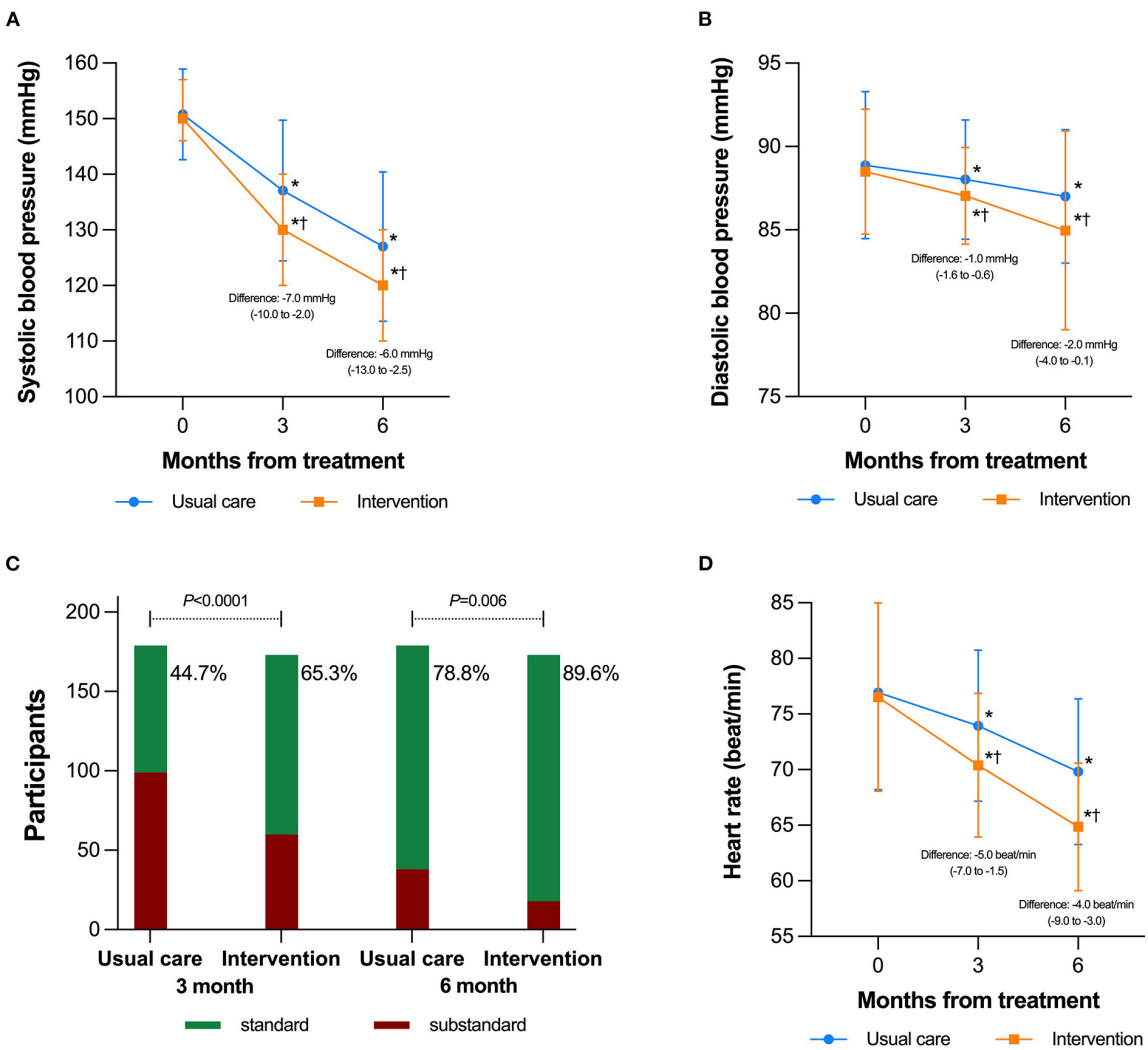
Blood pressure changes during the study period by group. **(A)** Mean values and standard deviations in systolic blood pressure (mmHg) among all participants; **(B)** Mean values and standard deviations in diastolic blood pressure (mmHg) among all participants; **(C)** Percentage of subjects achieving treatment goal; **(D)** Changes in heart rate, which were presented as mean and standard deviations (error bar). *P < 0.05 within group vs. baseline value;^†^*P* < 0.05 compared the intervention group with the usual care group.

Overall, greater BP lowering effects were observed after intervention from the numerical BP results. As for SBP, intervention group showed differences of −7.0 (−10.0 to −2.0) mmHg at Month 3 and −6.0 (−13.0 to −2.5) mmHg at Month 6 compared to UC group. The absolute reduction in DBP was also larger with intervention: −3.0 (−4.0 to −2.0) and −7.0 (−11.0 to −5.5) mmHg at Month 3 and 6, respectively, as shown in Figure 2. Besides, intervention showed difference on HR decrease with −5.0 (−7.0 to −1.5) and −4.0 (−9.0 to −3.0) beats/min as compared to UC group at Month 3 and 6, respectively.

Specifically, there was significant difference in the proportion of subjects who achieved target BP during the 3- and 6-month follow-ups. As shown in Figure 2C, 65.3 (*n* = 113/173) and 44.7% (*n* = 80/173) of patients achieved BP goals after receiving intervention and UC, respectively at month 3 (OR = 1.461, 95% CI = 1.202–1.778, *P* < 0.001), while the proportion of patients achieving target BP for intervention and UC group was 89.6 (*n* = 155) and 78.8% (*n* = 141) (OR = 1.137, 95% CI = 1.038–1.246, *P* = 0.006).

### 3.3. Adherence with anti-hypertensive medications

**Figure 3:**
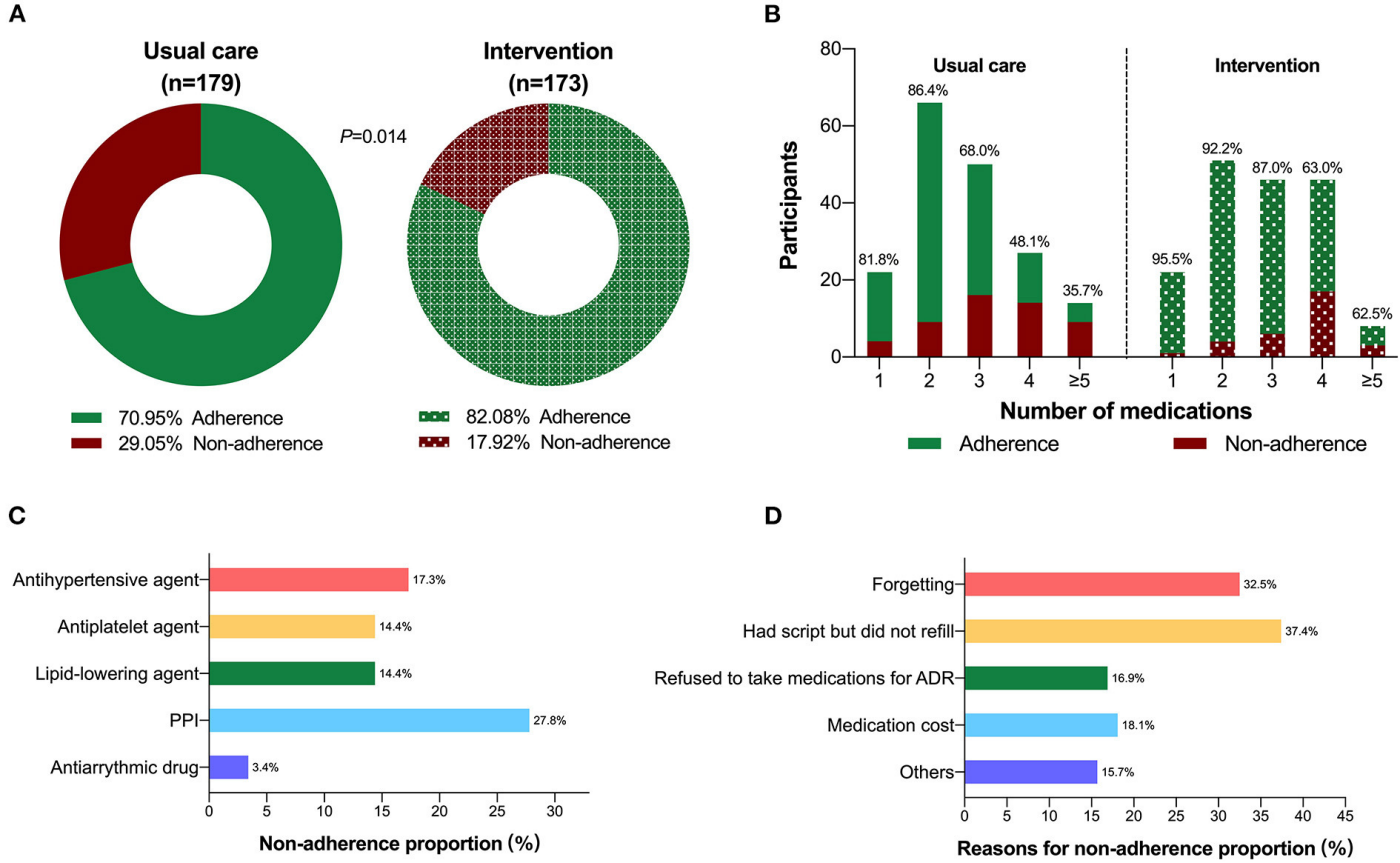
Rates of total adherence stratified by group. **(A)** Number of medication prescribed **(B)**; **(C)** Drug nonadherence by different medication classes in all cohort; **(D)** Reasons for non-adherence among non-adherent patients.

Throughout the treatment period, 142 (82.1%) persons in intervention group and 127 (71.0%) persons in usual care group reported full adherence (*P* = 0.014) (Figure 3A). Overall, medication adherence dropped as the number of prescribed medications increased (Figure 3B). Participants exhibited the highest compliance rates to antiarrhythmic agents of over 95%, while the lowest rate of below 75% was reported with PPI (Figure 3C). The most common causes of non-adherence were reported as having script but not refilling (37.4%) and forgetting (32.5%) (Figure 3D).

### 3.4. Medication related interventions

During the follow-up period, the mean number of active outpatient revisits attended by participants was 2.22 ± 1.09 and 3.30 ± 1.37 in the intervention and usual care groups, respectively (*P* < 0.0001). In addition to the regular check-ups, 299 interventions were provided according to patient questioning online or at the clinic. A comparison of medication therapy interventions between non- and pharmacist intervention groups was shown in Supplementary Figure 2. Compared to usual care group, pharmacist-conducted patients had more concerns about enhancing medication adherence (19.1 vs. 7.3%, *P* = 0.001). Participants who received usual care were more likely to raise questions on drug-drug interaction, side effects and discontinuation of therapy (21.8 vs. 12.7%, *P* = 0.025; 11.2 vs. 5.2%, *P* = 0.042; 11.7 vs. 3.5%, *P* = 0.004, respectively).

### 3.5. Clinical outcomes assessments

**Figure 4:**
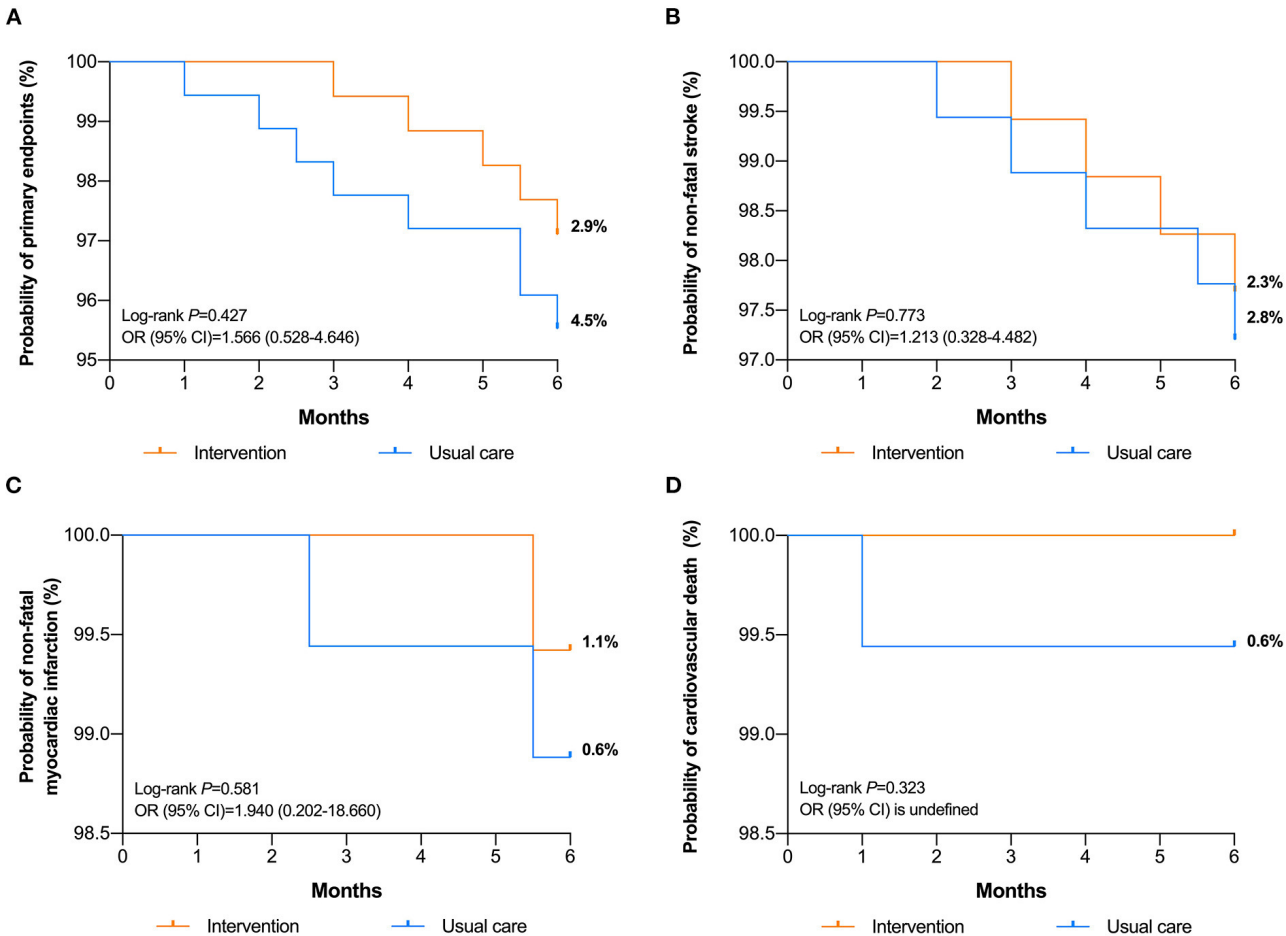
Kaplan-Meier cumulative curves for **(A)** composite of events, **(B)** non-fatal stroke, **(C)** non-fatal myocadiac infarction, and **(D)** cardiovascular death between intervention and UC groups.

There were 8 (4.5%) and 5 (2.9%) participants experiencing events as a composite of non-fatal stroke, non-fatal myocardial infarction and cardiovascular death in intervention and UC groups, respectively. As illustrated in Figure 4A, cumulative Kaplan-Meier results demonstrated that patients who belonged to UC group were more likely to experience major adverse cardiovascular events, but the difference was not statistically significant (*P* = 0.427, OR = 1.566, 95%CI: 0.528–4.646). Among all the events, 5 patients in the UC group and 4 patients in the intervention group developed non-fatal stroke (Figure 4B, *P* = 0.773, OR = 1.213, 95%CI: 0.328–4.482). With regards to non-fatal myocadiac infarction, cumulative incidence was similar between UC (*n* = 2) and intervention (*n* = 1) groups (*P* = 0.581, OR = 1.940, 95%CI: 0.202–18.660), as demonstrated in Figure 4C. During the study period, one subject from UC group died due to cardiovascular causes (Figure 4D).

## 4. Discussion

This observational study elucidated the advantage of pharmacist-led telemedicine on antihypertensive pharmacotherapy during the COVID-19 Pandemic. Our findings highlighted that telemedicine could significantly reduce BP and improved medication adherence at the established endpoint of 6 months. Pharmacist-led telemedicine may prove effective in reducing cardiovascular events in terms of stroke, non-fatal myocardial infarction and cardiovascular death as compared to regular outpatients with a longer duration of intervention.

Providing primary healthcare during COVID-19 pandemic has brought a huge challenge mainly due to inadequate availability of personal protective equipment and high risks of infection from patients and medical practitioners for healthcare service providers (17–19). Telemedicine minimizes in-person communication and reducing face-to-face contact among clinicians and patients (20), which was first officially recommended in 2019 Chinese guidelines for the management of hypertension in the elderly and compliant with the Program for a Healthy China 2030 (21, 22). Currently, pharmacists are playing important role in patient-centered model for hypertension care which required a higher demand on interprofessional collaboration (23). Pharmacists might expand their medication interventions and provide remote services for patients by means of telemedicine. In this study, pharmacist-led telemedicine reduced the patients' offline healthcare consultations, leading to a lower risk of Covid-19 exposure, as well as the reduction of time and cost.

Our study showed that the BP reduction levels following pharmaceutical telemedicine intervention was larger than that in UC groups, either in 3 or 6 months. Telemedicine technology is widely available, inexpensive and widely accepted by doctors and patients (24). A randomized controlled trial about Home and Online Management and Evaluation of Blood Pressure demonstrated the digital intervention resulted in better BP control than UC (12). The improvement could be achieved by overcoming barriers to medication adherence in the management of hypertension. As for most cases, these interventions studies were team-based managements with pharmacist-led care (25, 26). One meta-analysis illustrated an average 10/4 mmHg decrease of SBP/DBP, and an absolute proportion within target BP improved by 20% after receiving telemedicine care (27). Another recent study reported a well-controlled BP at baseline (15 mmHg lower at 12 months follow-ups with a significant difference than UC) (28). Based on the current evidence, the most common practice model of telepharmacy utilized outside licensed pharmacy was scheduled health care interventions *via* WeChat for management of cardiovascular disease, mostly hypertension and diabetes (29). A previous study documented a promotion of adherence to BP monitoring and telemedicine management visits after telepharmacy intervention, thus revealing that this approach is feasible and effective (20). Telemedicine intervention by pharmacist can effectively improve the self-efficacy and medication compliance of patients with hypertension, the drug treatment management can especially reflect the professional value of pharmacists and is of great value to the management of hypertension. In addition, it also plays an important role in helping specialist pharmacists provide convenient, patient-centered pharmacy services.

Based on our findings, the benefits of pharmacist-led intervention became apparent with increasing numbers of medications. Pharmacist intervention might improve patients' understanding on medications, especially for polypharmacy patients, leading to better adherence (30). Our study also found patients exhibited worst adherence in taking PPIs, probably related with neglect and insufficient knowledge, indicating the need of more instruction from pharmacist in this aspect in the future. When regarding the reasons for non-adherence, patients not refilling medications despite having prescriptions and forgetting to take medications occupied more than half proportion, followed by fear of drug induced ADR, similar with one published study (31). Thus, pharmacist would better design a remote reminder tool to avoid omitting dose and provide guidance on precautions to cope with ADRs. Moreover, another retrospective study showed the initial pharmacist intervention could be considered most important, as patients completing the initial intervention were less likely to discontinue follow-up and more likely to be adherent (32).

Our study provided additional benefits of the pharmacist intervention. It seemed that pharmacist-conducted patients were more willing to enhance medication adherence (19.1 vs. 7.3%, *P* = 0.001). These positive findings could be replicated in the previous study which demonstrated that pharmacist-led medication counseling could achieve better optimal BP control and enhance compliance (33, 34). Although, another study had the opposite conclusion that there were no significant differences in medication compliance by pharmacist counseling, which may be attributed to the selection patients with low medication adherence in the study (35). Besides, we provided additional results associated with medication related interventions. Participants who received usual care were more likely to raise questions on drug-drug interaction, side effects and discontinuation of therapy (21.8 vs. 12.7%, *P* = 0.025; 11.2 vs. 5.2%, *P* = 0.042; 11.7 vs. 3.5%, *P* = 0.004, respectively). These results were partly supported by a previous pharmacist-led drug counseling study (36), which have evaluated 70–80% of patients were concerned about the solutions of adverse reactions and 50–60% focused on drug interactions. In a word, pharmacist-led interventions have the potential to magnify the health benefits of medications.

Although the incidence of major adverse cardiovascular events pointed in the direction in favor of pharmacists' intervention, no significant differences were found. Previous meta-analysis finds that good compliance to cardiovascular medications decrease 20% risk of cardiovascular events (37). Achievement of long-term BP target value also suggests better outcomes (38). The effects on adherence and BP targets reached in intervention group were not translated into remarkable decrease of cardiovascular events. The main reasons for the non-significant results were attributed to the small sample sizes and short follow-up time. Further large, long-term follow-up trials are required to evaluate the effect on composite endpoints.

Our study had some limitations. Firstly, this was a prospective analysis with relatively small sample size and further randomized controlled trials are needed to confirm the conclusions. Secondly, the adherence was based on self-report in this study, remaining a degree of subjectivity. Biological measurement and a validated daily reporting system considering medication refill rate are the best ways to measure medication adherence, which were not available at this time. Thirdly, this study was not placebo-controlled, therefore the findings on symptomatic status are subjective to a placebo effect. Finally, the follow-up was too short to detect long-term differences in clinical cardiovascular adverse outcomes.

## 5. Conclusion

In summary, pharmacist-led telemedicine for hypertension management had led to better BP control and medication adherence improvement than UC during COVID-19 epidemic, resulting in a reduction of overall adverse cardiovascular events occurrence. The further work is to realize clinical benefits for chronic illness care with this implementation strategy during COVID-19 epidemic.

## Data availability statement

The raw data supporting the conclusions of this article will be made available by the authors, without undue reservation.

## Ethics statement

The studies involving human participants were reviewed and approved by the Ethics Committee of Zhongshan Hospital. The patients/participants provided their written informed consent to participate in this study.

## Author contributions

XiaoyeL and JH: writing, original draft preparation, and investigation and data curation. YY: investigation and formal analysis and software. CZ: methodology. ZW: software. XiaoyuL: conceptualization. QL: supervision and writing—reviewing and editing. All authors contributed to the article and approved the submitted version.
