# Supplementary material for: Evaluation of pharmacist-led telemedicine medication management for hypertension established patients during COVID-19 pandemic: A pilot study
Source: Front Public Health. 2022 Dec 16;10:1091484. doi: 10.3389/fpubh.2022.1091484 (PMC9800920; doi:10.3389/fpubh.2022.1091484)

**Supplementary Materials**

Supplementary figures and table are listed as below:

**Supplementary Figure 1**. Schematic representation of pharmacist-led telemedicine and home BP monitoring.


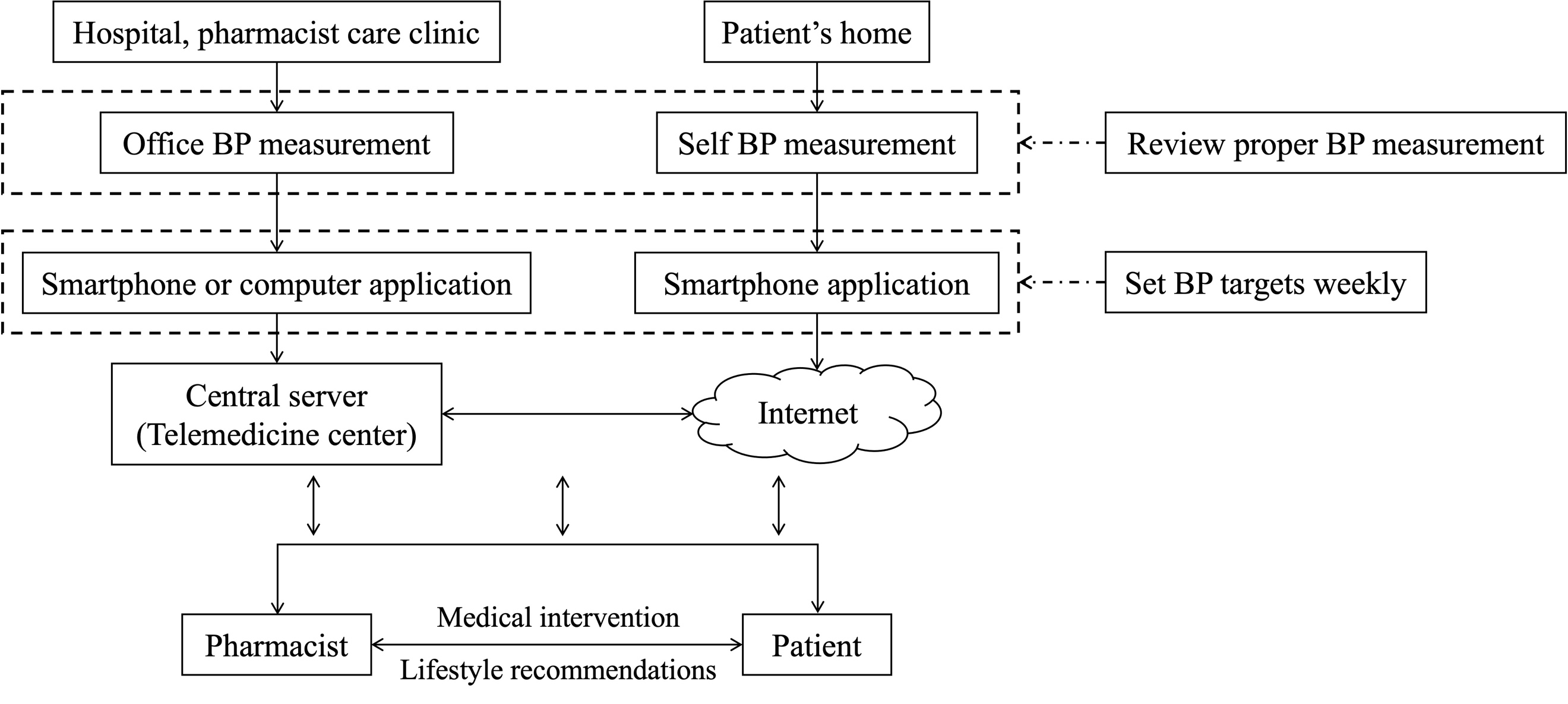


BP, blood pressure.

**Supplementary Table 1**. blood pressure medication guidance

| **1. Principles of antihypertensive pharmacotherapy**  The fundamental goal of hypertension treatment is to control blood pressure and reduce the risk of hypertension-related cardiovascular and cerebrovascular diseases and death. Medication guidelines recommend lifestyle improvements, drug selection and other risk factor management based on the patient’s blood pressure and overall risk level. |
| --- |
| **2. Administration**  Please follow the doctors’ or clinical pharmacists’ instructions.  Take medicine at the same time every day.  Priority access to long-acting preparations that are administered once a day and have a 24-hour antihypertensive effect. |
| **3. Blood pressure monitoring**  Participants were advised to take blood pressure readings twice a day. |
| **4. Adverse reactions and corresponding preventive measures**  Diuretic: Electrolyte disorders, abnormal glucose metabolism, hyperuricaemia, etc. Notes: Electrolyte, blood glucose and uric acid should be tested during treatment. Most patients need combination drug therapy.  ACEI/ARB: Angioedema, hyperkalemia, facial flushing, cough, etc. Note: Blood potassium, creatinine and EGFR should be monitoredd during ACEI/ARB treatment.  CCB: Rapid heart rates, oedema, facial flushing, hypotension, etc. Note: Medical advice should be sought and discontinue medicine in severe cases.  β-blockers: Tachycardia, atrioventricular block, asthma aggravation, etc. Note: Monitor blood pressure and heart rate regularly. Excessively low heart rate is forbidden.  α-blockers: Orthostatic hypotension, rapid heart rates, gastrointestinal distress, etc. Note: It is recommended that patients take their initial medication before bedtime. |
| **5. Other attentions**  Balanced diet. Avoid greasy and salty foods.  Regular lifestyle. Avoid staying up late and being tired.  Quit smoking and set limit on alcohol.  Take antihypertensive medicine and monitor blood pressure regularly. Avoid dose adjustion ordrug withdrawal without authorization. |
| 1. Laboratory tests   ALT, Hb, PLT, eGFR, LDL-C, HbA1c, APTT, PT, TT, FIB, D-D, INR, CK-MB, NT-proBNP |

ALT, alanine aminotransferase; Hb, hemoglobin; PLT, platelet count; eGFR, estimated glomerular filtration rate, calculated with the CKD-EPI Equation; LDL-C, low-density lipoprotein cholesterol; HbA1c, glycosylated hemoglobin type A1c; APTT, activated partial thromboplastin time; PT, prothrombin time; TT, thrombin time. FIB, fibrinogen. D-D, d-dimer; INR, international normalized ratio; CK-MB, creatine kinase-MB; NT-proBNP, N-terminal pro-B-type natriuretic peptide.

**Supplementary Figure 2.** The comparison of medication related interventions between intervention and UC groups


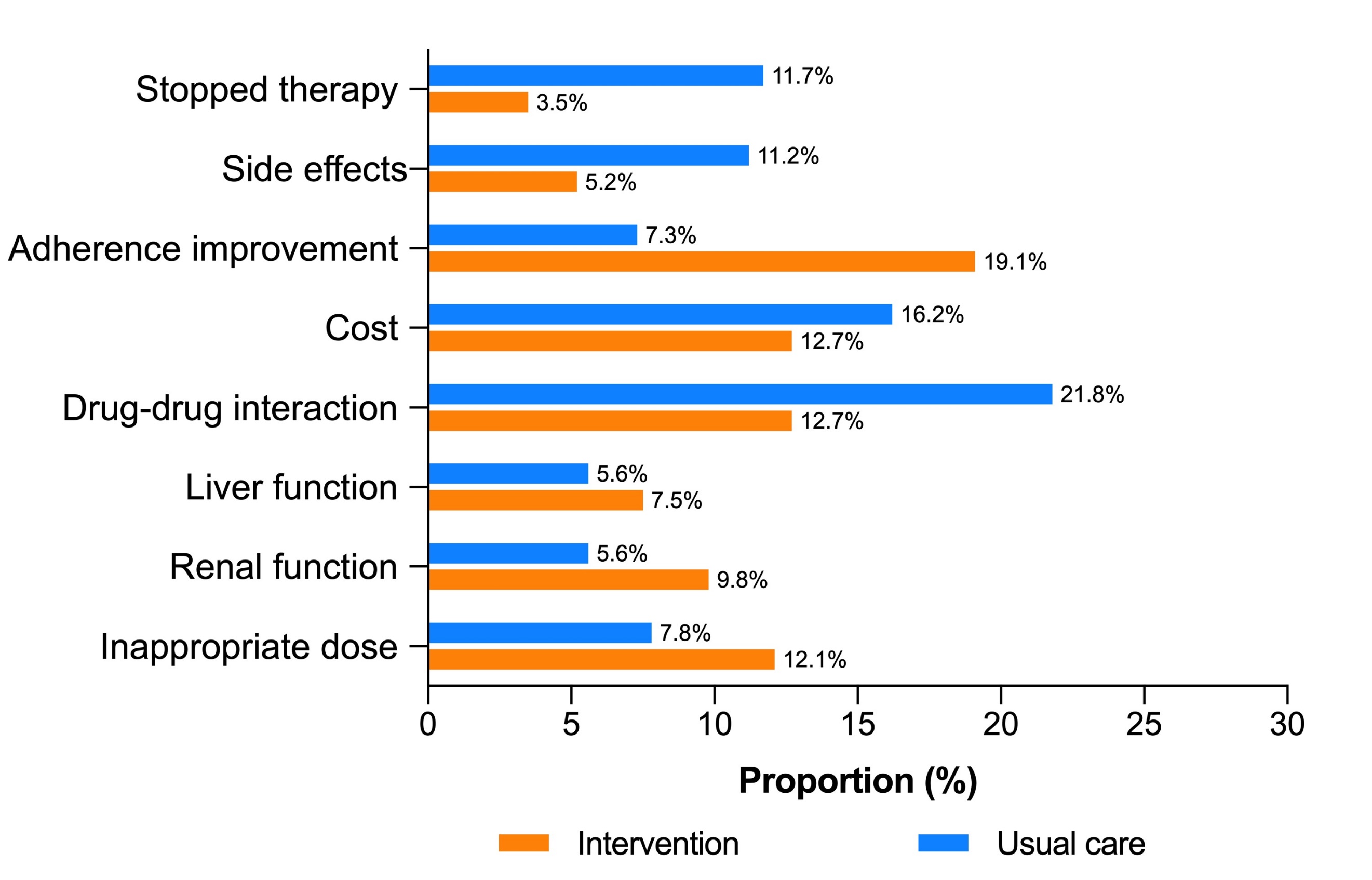

Supplement: Supplementary file 1 [file Data_Sheet_1.docx]
